# Supplementary material for: Adjuvant chemotherapy compared with observation in patients with resected biliary tract cancer: A systematic review and meta-analysis of randomized controlled trials
Source: PLoS One. 2025 Apr 23;20(4):e0295583. doi: 10.1371/journal.pone.0295583 (PMC12017477; doi:10.1371/journal.pone.0295583)
Supplement: S2 Table — (DOCX) [file pone.0295583.s002.docx]

**S2 Table. The MEDLINE (OVID format) search strategy used in this meta-analysis.**

| 1. randomized controlled trial.pt. | 21. (bile duct adj5 tumor$).mp |
| --- | --- |
| 2. controlled clinical trial.pt. | 22. exp gallbladder cancer/ |
| 3. randomized.ab. | 23. (gallbladder adj5 cancer$).mp. |
| 4. placebo.ab. | 24. (gallbladder adj5 neoplasm$).mp |
| 5. drug therapy.fs. | 25. (gallbladder adj5 carcinoma$).mp |
| 6. randomly.ab. | 26. (gallbladder adj5 tumor$).mp |
| 7. trial.ab. | 27. exp cholangiocarcinoma/ |
| 8. groups.ab. | 28. exp ampullary cancer/ |
| 9. or/1-8 | 29. or 12-28 |
| 10. humans.sh. | 30. exp drug therapy/ |
| 11. 9 and 10 | 31. chemothera$.tw. |
| 12. exp biliary tract cancer/ | 32. drug therap$.tw. |
| 13. (biliary tract adj5 cancer$).mp. | 33. antineoplastic$.tw. |
| 14. (biliary tract adj5 neoplasm$).mp | 34. or/30-33 |
| 15. (biliary tract adj5 carcinoma$).mp | 35. resect$.tw |
| 16. (biliary tract adj5 tumor$).mp | 36. opera$.tw |
| 17. exp bile duct cancer/ | 37. or/35-36 |
| 18. (bile duct adj5 cancer$).mp. | 38. 11 and 29 |
| 19. (bile duct adj5 neoplasm$).mp | 39. 34 and 37 |
| 20. (bile duct adj5 carcinoma$).mp | 40. 38 and 39 |

pt: publication type. ab: abstract. fs: floating subheading. sh: subheading. adj: adjacent. mp: indicates a search of title, original title, abstract, name of substance word and subheading word. tw: text word. $: truncation operator.
