# Supplementary material for: Adjuvant chemotherapy compared with observation in patients with resected biliary tract cancer: A systematic review and meta-analysis of randomized controlled trials
Source: PLoS One. 2025 Apr 23;20(4):e0295583. doi: 10.1371/journal.pone.0295583 (PMC12017477; doi:10.1371/journal.pone.0295583)
Supplement: S1 Appendix — (DOCX) [file pone.0295583.s003.docx]

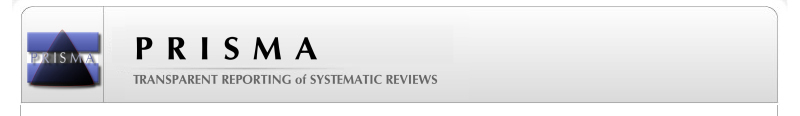
**PRISMA 2009 Flow Diagram**

Full-text articles excluded (n=45)

23 phase I trials

12 not-controlled design

9 retrospective studies

1 for periampullary carcinoma

Records excluded (n = 275)

157 reviews

23 for chemoradiotherapy or radiotherapy

22 not compared with observation

5 for immunotherapy

10 for targeted therapy

58 case reports or others

Full-text articles assessed for eligibility
(n = 50)

Studies included in qualitative synthesis
(n = 5)

Studies included in quantitative synthesis (meta-analysis)
(n = 5)

Records screened
(n =325)

Records after duplicates removed
(n = 325)

Additional records identified through other sources
(n = 0)

## Identification

## Eligibility

## Included

## Screening

Records identified through database searching
(n = 382)
