# Supplementary figures and images for: Adjuvant chemotherapy compared with observation in patients with resected biliary tract cancer: A systematic review and meta-analysis of randomized controlled trials
Source: PLoS One. 2025 Apr 23;20(4):e0295583. doi: 10.1371/journal.pone.0295583 (PMC12017477; doi:10.1371/journal.pone.0295583)

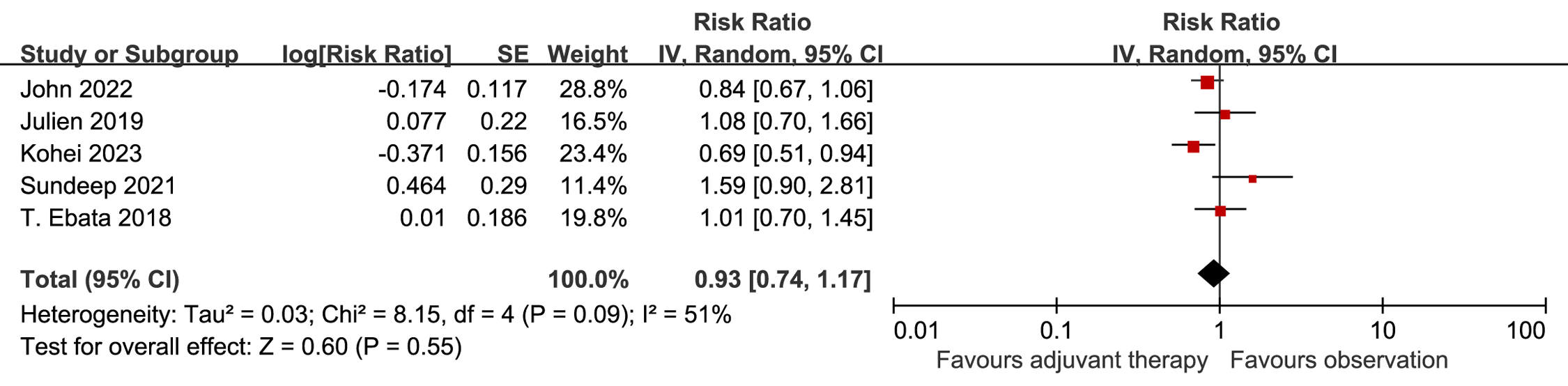

Supplement: S1 Fig — SE: Standard error. CI: Confidence interval. IV: Inverse variance. (TIF) [file pone.0295583.s004.tif]

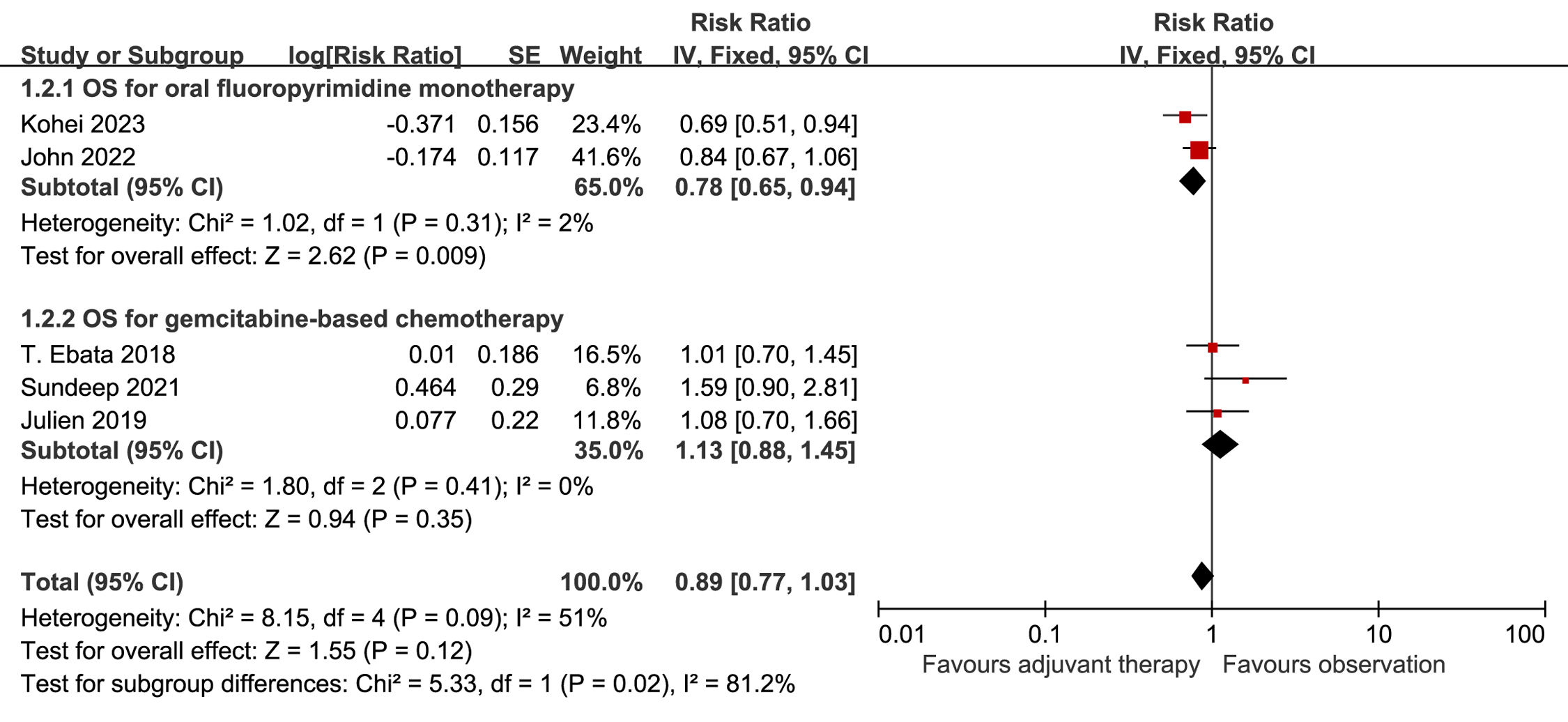

Supplement: S2 Fig — SE: Standard error. CI: Confidence interval. IV: Inverse variance. (TIF) [file pone.0295583.s005.tif]

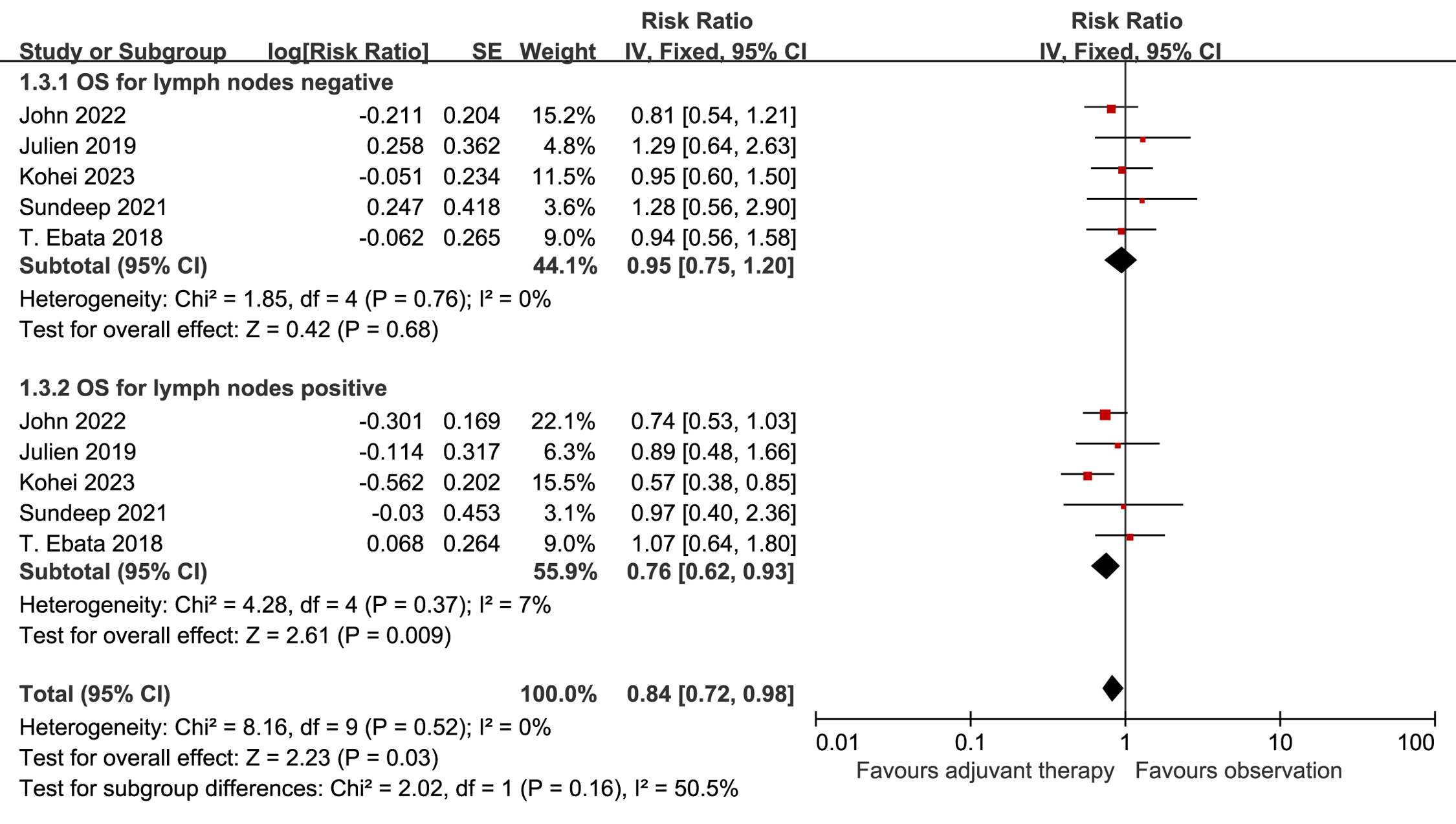

Supplement: S3 Fig — SE: Standard error. CI: Confidence interval. IV: Inverse variance. (TIF) [file pone.0295583.s006.tif]

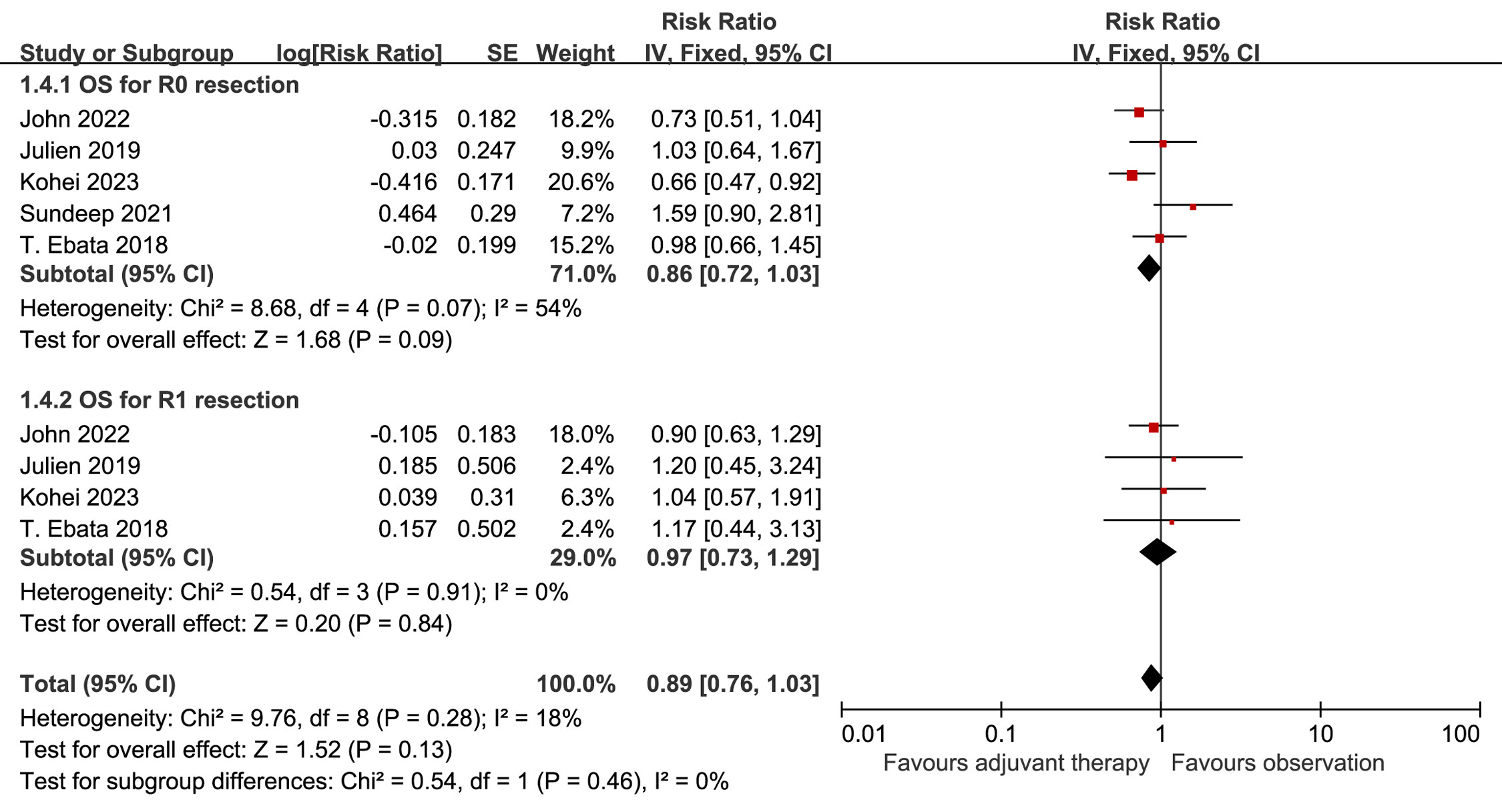

Supplement: S4 Fig — SE: Standard error. CI: Confidence interval. IV: Inverse variance. (TIF) [file pone.0295583.s007.tif]

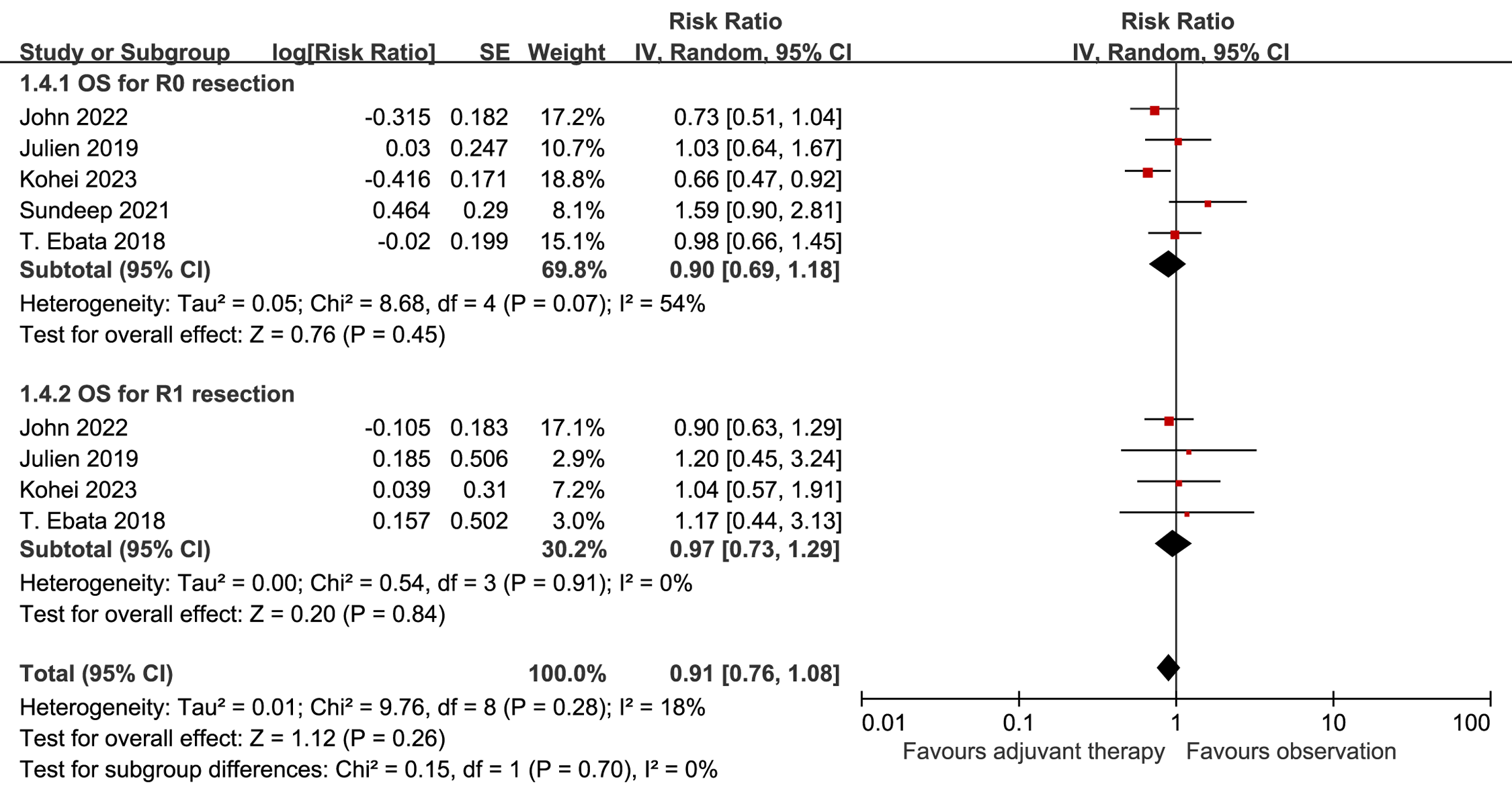

Supplement: S5 Fig — SE: Standard error. CI: Confidence interval. IV: Inverse variance. (TIF) [file pone.0295583.s008.tif]

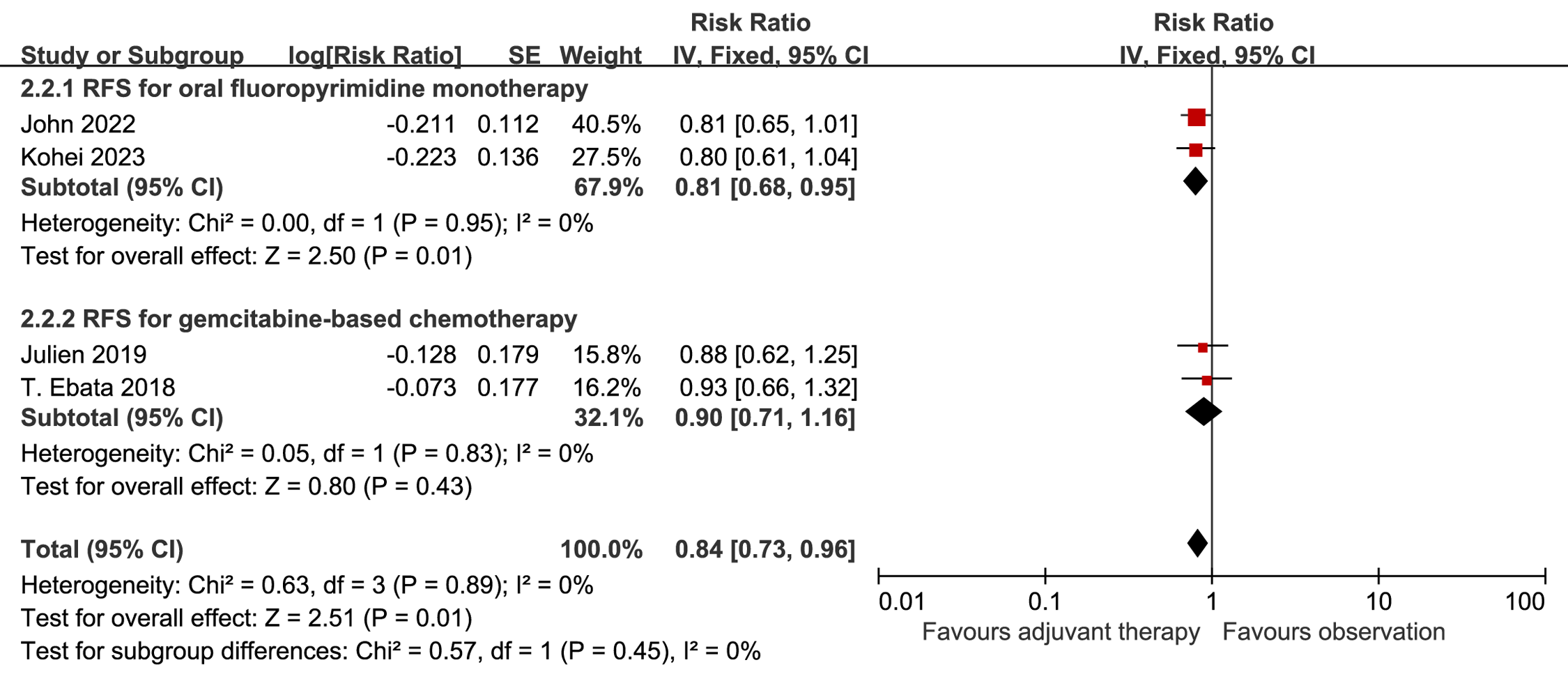

Supplement: S6 Fig — SE: Standard error. CI: Confidence interval. IV: Inverse variance. (TIF) [file pone.0295583.s009.tif]

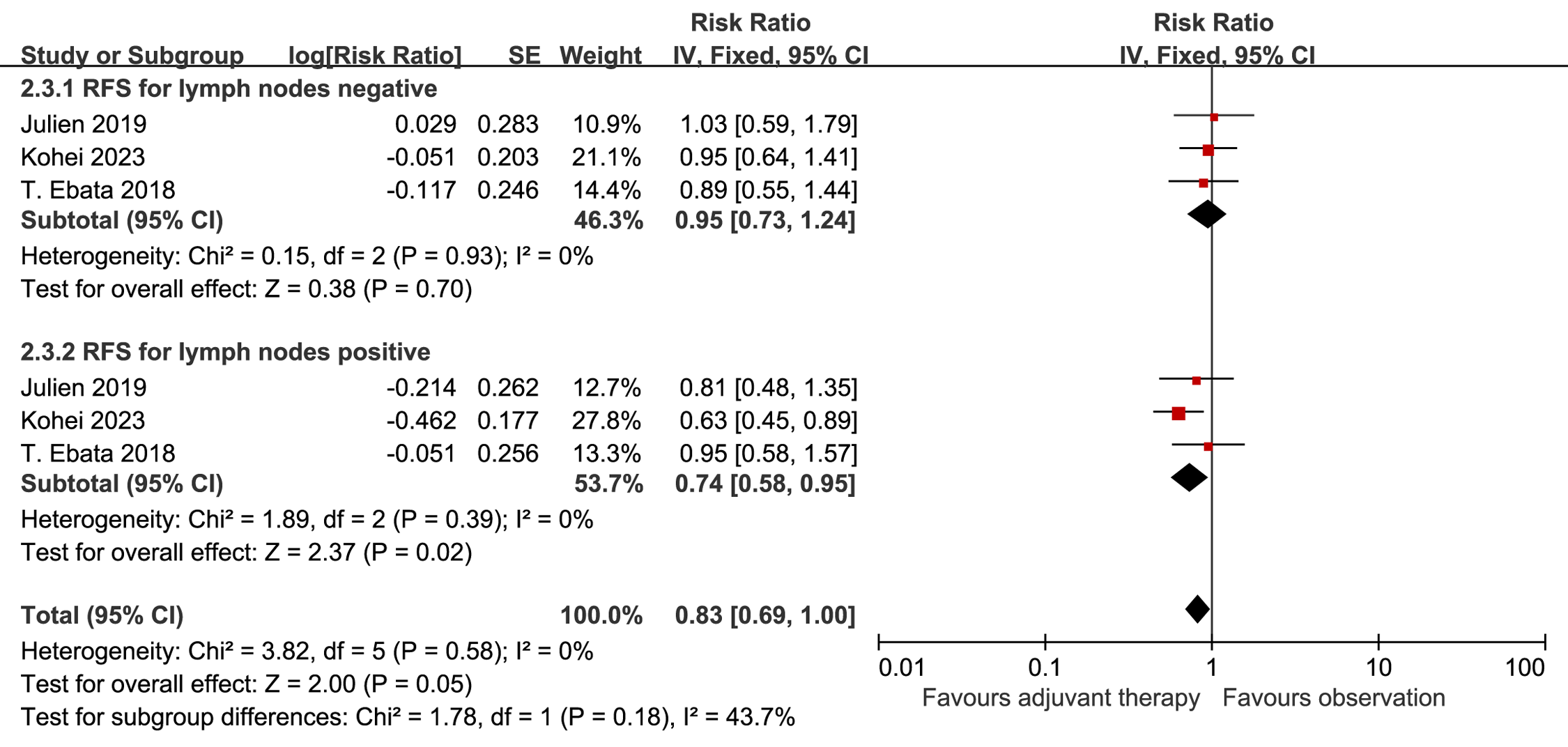

Supplement: S7 Fig — SE: Standard error. CI: Confidence interval. IV: Inverse variance. (TIF) [file pone.0295583.s010.tif]

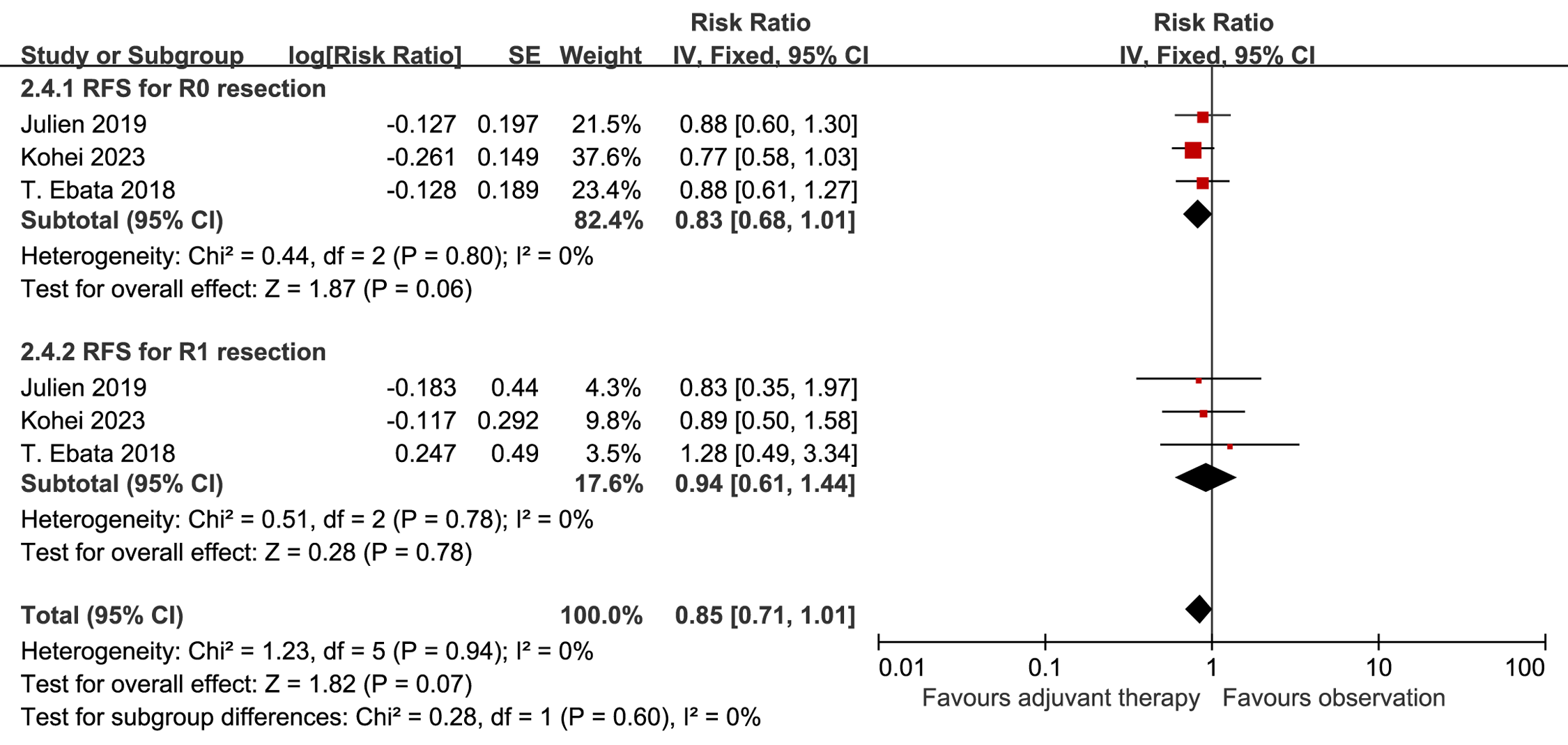

Supplement: S8 Fig — SE: Standard error. CI: Confidence interval. IV: Inverse variance. (TIF) [file pone.0295583.s011.tif]

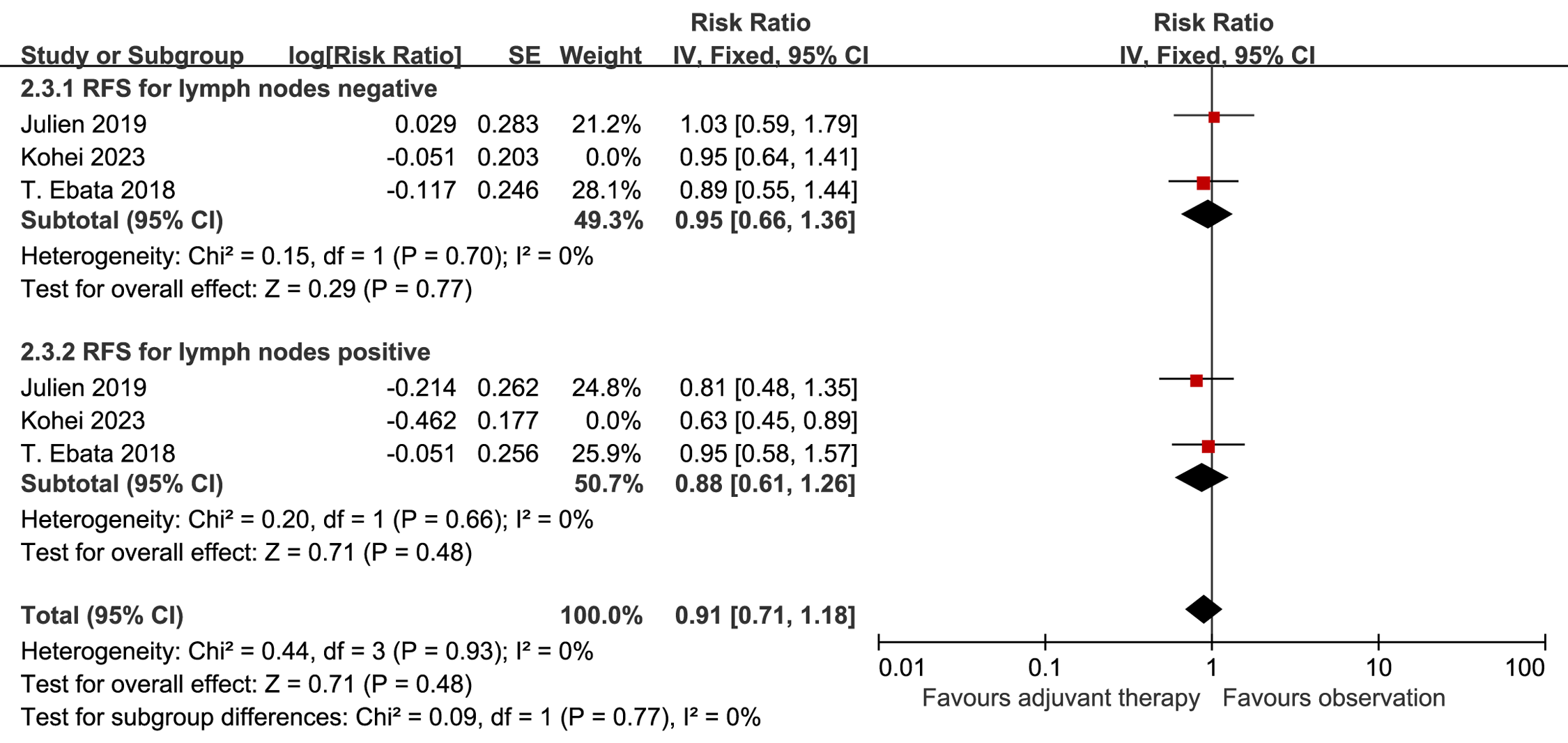

Supplement: S9 Fig — SE: Standard error. CI: Confidence interval. IV: Inverse variance. (TIF) [file pone.0295583.s012.tif]
