# Supplementary material for: Adjuvant chemotherapy compared with observation in patients with resected biliary tract cancer: A systematic review and meta-analysis of randomized controlled trials
Source: PLoS One. 2025 Apr 23;20(4):e0295583. doi: 10.1371/journal.pone.0295583 (PMC12017477; doi:10.1371/journal.pone.0295583)
Supplement: Attachment — Submitted filename: Response to Reviewers.docx [file pone.0295583.s014.docx]

December 16, 2023

Reply for manuscript “Adjuvant chemotherapy compared with observation in patients with resected biliary tract cancer: a systematic review and meta-analysis of randomized controlled trials”

 **Reply to editor:**

Dear editor:

Thank you for providing us with the opportunity to submit the revised manuscript. We would like to express our sincere gratitude to all the reviewers for their constructive comments. Based on these helpful comments, we have extensively revised the manuscript by correcting mistakes and supplementing the required materials to enhance its quality. Additionally, we have ensured that the manuscript adheres to PLOS ONE's style requirements.

Journal Requirements:

When submitting your revision, we need you to address these additional requirements.

1. Please ensure that your manuscript meets PLOS ONE's style requirements, including those for file naming. The PLOS ONE style templates can be found at [https://journals.plos.org/plosone/s/file?id=wjVg/PLOSOne_formatting_sample_main_body.pdf and](https://journals.plos.org/plosone/s/file?id=wjVg/PLOSOne_formatting_sample_main_body.pdf%20and) <https://journals.plos.org/plosone/s/file?id=ba62/PLOSOne_formatting_sample_title_authors_affiliations.pdf>

Answer: We have carefully reviewed our manuscript to ensure that it meets PLOS ONE's style requirements.

2. Please include a caption for figure 1.

Answer: We have added the caption for figure 1. Line 149.

3. Please include captions for your Supporting Information files at the end of your manuscript, and update any in-text citations to match accordingly. Please see our Supporting Information guidelines for more information: <http://journals.plos.org/plosone/s/supporting-information>.

Answer: We have added captions for Supporting information in the end of our manuscript. Line 341-343.

**Reply to reviewers:**

**Dear reviewer:**

We greatly appreciate your time and helpful comments on our manuscript. These comments are valuable and greatly help our manuscript. We have considered each comment and replied individually and marked the line number in the revised manuscript where we track the change. We hope you are more satisfied with the revised version. We would be happy to make further revisions to the manuscript.

**Reply to reviewer 1:**

Reviewer #1: Adjuvant chemotherapy compared with observation in patients with resected biliary tract cancer: a systematic review and meta-analysis of randomized controlled trials.
Conclusions: To conclude, adjuvant chemotherapy had the potential to offer advantages in patients with resected BTC. How long has adjuvant chemotherapy extended the survival period?

Answer: We have added the survival benefit scenarios to the Discussion section. Line 259-263.

**Reply to reviewer 2:**

Reviewer #2: The study assesses a current, timely topic in biliary tract cancer. An impressive number of patients and studies have been included.

A linguistic revision is required.

Answer: The manuscript has been linguistically polished, especially in the background and discussion sections.

We believe this article is suitable for publication in the journal although some revisions are needed. The main strengths of this paper are that it addresses an interesting and very timely question and provides a clear answer, with some limitations. Certainly, the authors should better highlight the limitations of the current paper.

Answer: We have enriched the limitation part of our manuscript by including a more comprehensive discussion. Line 327-332.

- The background of the changing scenario of medical treatment in BTC should be better discussed, and some recent papers regarding this topic should be included ( PMID: 36633661; PMID: 33592561; PMID: 33756174 ; PMID: 35031442 ).

Answer: We have enriched our discussion part of the manuscript based on the recent papers, and added the papers (PMID: 36633661; PMID: 33592561; PMID: 33756174) in the reference. Line 251-252. Line 299-301. Line 313-315.
